# Stronger Associations of Body Mass Index and Waist Circumference with Diabetes than Waist-Height Ratio and Triglyceride Glucose Index in the Middle-Aged and Elderly Population: A Retrospective Cohort Study

**DOI:** 10.1155/2022/9982390

**Published:** 2022-02-26

**Authors:** Kun He, Wenli Zhang, Xueqi Hu, Hao Zhao, Rui Song, Kaizhi Bai, Wenlong Shi, Songhe Shi, Zhan Shi, Mingshu Yan

**Affiliations:** ^1^Department of Epidemiology and Health Statistics, College of Public Health, Zhengzhou University, Zhengzhou, Henan, China; ^2^Department of Pharmacy, Zhengzhou People's Hospital, Zhengzhou, Henan, China; ^3^College of Nursing and Health, Zhengzhou University, Zhengzhou, Henan, China

## Abstract

**Background:**

It remains controversial whether body mass index (BMI), waist circumference (WC), waist-to-height ratio (WHtR), or triglyceride glucose (TyG) index has a stronger association with diabetes. The aims of the study were to compare the magnitude of associations of four indicators with diabetes risk.

**Methods:**

Data collected from annual health examination dataset in the Xinzheng during 2011 and 2019. A total of 41,242 participants aged ≥ 45 years were included in this study. Cox proportional hazard regression models were used to examine associations between the four indicators and diabetes risk.

**Results:**

After 205,770 person-years of follow up, diabetes developed in 2,472 subjects. Multivariable-adjusted hazard ratios (HRs) and 95% confidence intervals (CIs) of diabetes (highest vs reference group) were 1.92 (1.71–2.16) for BMI, 1.99 (1.78–2.23) for WC, 1.65 (1.47–1.86) for WHtR, and 1.66 (1.47–1.87) for TyG, respectively. In addition, the risk of diabetes increased with baseline BMI (HR: 1.30; 95% CI: 1.25, 1.35) and TyG (HR: 1.25; 95% CI: 1.20, 1.30), but the lowest HR was 0.78 (95% CI 0.65–0.92) when WC was approximately 72 cm, and 0.85 (95% CI 0.72–0.99) when WHtR was approximately 0.47 in women. In joint analyses, the highest risk was observed in participants with a high BMI combined with a high WC (HR: 2.26; 95% CI: 1.98, 2.58).

**Conclusions:**

In middle-aged and elderly Chinese population, BMI and WC were more strongly associated with diabetes than WHtR or TyG, especially the combined effect of BMI and WC.

## 1. Introduction

As a major chronic noncommunicable disease, diabetes can cause retinal, renal, cardiovascular, and nervous system complications, increasing public health burden [1]. Currently, China has the largest number of people with diabetes (116 million in 2019), and it is estimated that the numbers will reach 140 million by 2035 [2]. However, previous studies have shown that half (50.1%) of people with diabetes do not suspect they have the disease before diagnosis [2]. Therefore, it is very important to identify high-risk individuals as early as possible and to reduce the global epidemic of diabetes via a simple, effective, and reproducible diagnostic index.

Previous literature has indicated that obesity, which is most commonly assessed by body mass index (BMI), waist circumference (WC), and waist-to-height ratio (WHtR), is one of the most important modifiable risk factors for type 2 diabetes mellitus (T2DM). Therefore, the prevention and screening of diabetes are recommended in individuals with obesity [3–5]. Although BMI is widely used to identify obesity, it does not reflect body fat distribution. The stronger association between abdominal fat and metabolic disorders has led some to suggest that anthropometric measures that describe the distribution of central fat, such as WC and WHtR, may be better predictors of diabetes than the general obesity indicator (BMI). Since WC does not take into account individual differences in body size and height, WHtR may represent an excellent predictor of diabetes. To our knowledge, insulin resistance (IR) forms a pivotal pathophysiological pathway in T2DM [6], and studies have shown that the triglyceride glucose (TyG) index can be used as an inexpensive and reliable surrogate indicator to identify IR, which is useful for the early identification of individuals at risk for T2DM [7, 8]. However, previous studies found that BMI was more strongly associated with diabetes risk, or there was no significant difference compared to other abdominal adiposity indicators in predicting diabetes risk [9, 10], while other studies showed that compared to BMI, WC, or WHtR had the highest predictive power for diabetes [3, 5]. In addition, other studies have shown that TyG was significantly associated with the development of diabetes, and its predictive effect for diabetes was better than WC and WHtR in women [8, 11].

Therefore, there is still considerable debate over which indicator is more strongly associated with diabetes risk. The goal of this study was to compare the association of BMI, WC, WHtR, and TyG with the risk of diabetes.

## 2. Methods

### 2.1. Study Design and Study Population

The present study was a population-based, retrospective cohort study. Data originated from annual residents' health records in Xinzheng City, Henan Province, Central China. The electronic health records of residents mainly include three parts, namely questionnaire survey, anthropometric, and laboratory measurements. From January 2011 to December 2019, the final study cohort included 130,580 subjects aged ≥ 18 years who had ≥ 2 physical examination data. From the 130,580 participants, we first excluded 68,073 participants who lacked laboratory data at both baseline and follow-up. Then we excluded those with T2DM at baseline (*n* = 12,087), or with incomplete baseline data on smoking, drinking, physical activity, height, weight, WC, systemic blood pressure (SBP), diastolic blood pressure (DBP) (*n* = 3,641), who < 45 years old at baseline (*n* = 580), and who died due to disease, accidents, or other causes at follow-up (*n* = 4,957). Finally, we had 41,242 participants to assess the risk of T2DM with BMI, WC, WHtR, and TyG index (Supplemental Figure 1). This study was approved by the Ethics Committee of Zhengzhou University, and written informed consent was obtained from all participants.

### 2.2. Baseline Examination and Data Collection

All participants completed a standardized questionnaire that included their sociodemographic characteristics (age, gender, and marital status), medical history (diabetes, hypertension, chronic obstructive pulmonary disease, coronary heart disease and stroke, etc.), smoking, drinking, and physical activity. Based on self-reported marital status, smoking, and drinking, participants were classified as follows: living with partner or without partner; nonsmokers or previous/current smokers; and never, occasionally, or daily drinkers. Physical activity was classified as never, occasionally, more than once a week or daily.

Physical examination was conducted by uniformly trained investigators using a standard protocol. Participants were asked to maintain a standing position while wearing light clothes without shoes and were measured twice. Then, the average was recorded. Height and weight were measured to the nearest 0.1 cm and 0.1 kg, respectively, and waist circumference was measured midway between the lower edge of the costal arch and the upper edge of the iliac crest to the nearest 0.1 cm under standardized conditions following a standard protocol. The BMI was calculated as weight (kg) divided by height squared (m), and WHtR was determined by WC (cm) divided by height (cm).

Resting heart rate (RHR) and blood pressure were measured twice after subjects had rested for at least 5 minutes in a seated position using an automatic sphygmomanometer (Omron HEM-7125, Kyoto, Japan) [12]. Blood samples were obtained after an overnight fast of at least 8 hours and were measured using an automatic biochemical analyzer (DIRUI CS380, Changchun, China). Laboratory parameters, including fasting plasma glucose (FPG), total cholesterol (TC), and triglycerides (TG), were used in this study. The TyG index was calculated as ln [TG (mg/dl) × FPG (mg/dl)/2] [13].

In this study, according to the Chinese guidelines for T2DM, diabetes was defined as: (1) self-reported doctor diagnosed diabetes, (2) fasting plasma glucose ≥ 7.0 mmol/L, or (3) current treatment with antidiabetic medication. Impaired fasting glucose (IFG) was defined as FPG ≥ 6.1 mmol/L and < 7.0 mmol/L^7^.

### 2.3. Statistical Analysis

Continuous variables are described using the median (interquartile range [IQR]) for skewed distribution, and categorical variables are expressed as frequency (%). Wilcoxon two-sample tests and Chi-square tests were used to compare the mean levels of baseline variables between subjects with and without diabetes.

Cox proportional hazard regression models were used to estimate the hazard ratios (HRs) and 95% confidence intervals (CIs) for BMI, WC, WHtR, and TyG in diabetes after confirming that the proportionality assumption was not violated. BMI was evaluated in the following 2 ways: (1) as four groups according to the Chinese BMI classification standard (underweight < 18.5 kg/m^2^, normal weight 18.5–23.9 kg/m^2^, overweight 24–27.9 kg/m^2^, and general obesity ≥ 28 kg/m^2^) [14] and (2) as a continuous variable. WC was evaluated in the following 3 ways: (1) as quartiles, (2) as a continuous variable, and (3) as two categories (noncentral obesity < 85 cm in women and < 90 cm in men; and central obesity ≥ 85 cm in women and ≥ 90 cm in men) [15]. WHtR was evaluated in the following 3 ways: (1) as quartiles, (2) as a continuous variable, and (3) as two categories (normal weight < 0.5; and central obesity ≥ 0.5) [16]. TyG index was evaluated in the following 2 ways: (1) as quartiles and (2) as a continuous variable. For WC, WHtR, and TyG indices, the lowest quartile was used as the reference group. The first model was unadjusted (model 1); model 2 was adjusted for age, gender, marital status, smoking, drinking, and physical activity; and model 3 was adjusted for variables included in model 2 in addition to RHR, SBP, DBP, and TC.

We used restricted cubic splines in the Cox models to characterize the dose-response association and test whether there is a nonlinear association of four indicators with diabetes risk. To compare the magnitude of risk estimates, we also calculated relative risks for per-SD changes in BMI, WC, WHtR, and TyG among the total population and subgroups and compared the global fitness of the models by Akaike's Information Criterion (AIC) [17], which is a measure of the trade-off between the goodness-of-fit of the regression model and the complexity of the model. The indicator with the smallest AIC was considered the best marker. In addition, we combined baseline BMI with WC to assess their combined effect on diabetes.

The sensitivity analysis was conducted to assess the robustness of the results after excluding participants with impaired FPG levels at baseline. The statistical analyses were performed using SAS 9.1 (SAS Institute, Cary, NC, USA). *P* < 0.05 for a two-sided test was considered statistically significant.

## 3. Results

### 3.1. Baseline Characteristics of Study Participants

The general characteristics of participants at baseline are described in Table 1. Data were analyzed for 41,242 middle-aged and elderly Chinese people (median age 63 years [interquartile range 61–68]). After 205,770 person-years of follow-up, diabetes developed in 2,472 participants; the overall incidence of diabetes was 12.01/1000 person-years. There were statistically significant differences in marital status, SBP, DBP, RHR, FPG, TG, BMI, WC, WHtR, and TyG index among patients with diabetes compared to those without diabetes (all *P* < 0.001).

### 3.2. Risk of Diabetes by Baseline BMI, WC, WHtR, and TyG Index

Table 2 presents HRs and 95% CIs for the associations between four indicators (BMI, WC, WHtR, and TyG index) and diabetes. In this study, BMI, WC, WHtR, and TyG were all positively and independently associated (model 1) with diabetes risk in a dose–response relationship (*P*_trend_ < 0.001). After further adjustment for confounders (model 2 and model 3), the associations remained significant. In model 3, the multivariable-adjusted HRs and 95% CIs for incident T2DM risk according to BMI levels at baseline were 0.69 (0.46–1.04), 1.00, 1.41 (1.29–1.54), and 1.92 (1.71–2.16) for BMI (18.5–23.9 [reference group]) (*P*_trend_ < 0.001). The cumulative risk of diabetes increased by baseline WC, WHtR, and TyG quartile and remained significant even after adjustment for potential confounding factors (HR [95% CI]: 1.19 [1.06–1.34], 1.34 [1.19–1.51], and 1.99 [1.78–2.23] for WC, 1.10 (0.98–1.24), 1.33 (1.18–1.49), and 1.65 (1.47–1.86) for WHtR, and 1.10 (0.97–1.25), 1.23 (1.09–1.39), and 1.66 (1.47–1.87) for TyG (all *P*_trend_ < 0.001), for quartiles 2, 3, and 4 versus quartile 1, respectively). Moreover, participants with abdominal obesity (WC ≥ 85 cm [women]/90 cm [men]) or high WHtR (≥ 0.5) had a 57% (95% CI: 1.45, 1.71) or a 39% (95% CI: 1.28, 1.51) higher risk of diabetes, respectively, compared to participants with low WC (< 85 cm [women]/90 cm [men]) or with low WHtR (< 0.5).

### 3.3. Restricted Cubic Spline Curves for Four Indicators and Diabetes Risk

**Figure 1:**
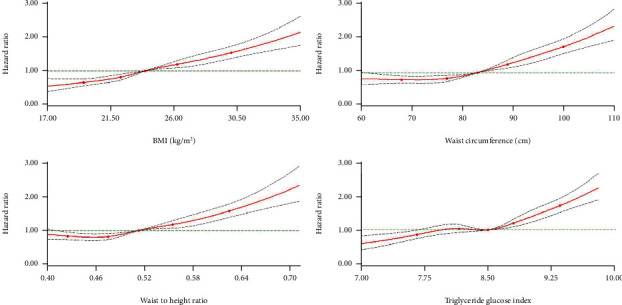
Hazard ratios for the association between four indicators (body mass index, waist circumference, waist to height ratio, and f triglyceride glucose index) and T2DM risk. The circles represent the points (5, 25, 50, 75, and 95 percentiles) where the nodes were placed. The region between the two dotted lines represents the 95% confidence intervals. The model was adjusted for age, gender, marital status, smoking, drinking, physical activity, RHR, RHR, SBP, DBP, and TC levels. Values were trimmed at less than 1st percentile and greater than 99th percentile of each indicator. SBP: systolic blood pressure; DBP: diastolic blood pressure; RHR: resting heart rate; TC: total cholesterol; BMI: body mass index; WC: waist circumference.

Figure 1 shows spline curves for the association of diabetes risk with the four indicators tested (BMI, WC, WHtR, and TyG index as a continuous variable), revealing approximately nonlinear associations for WC (*P* = 0.0013), WHtR (*P* = 0.0017), and TyG index (*P* = 0.001) but approximately log-linear associations for BMI (*P* = 0.408). The risk of diabetes increased with the increase of WC and WHtR in men, and TyG, but in women, the lowest HR was 0.78 (95% CI: 0.65–0.93) when WC was approximately 72 cm, and 0.85 (95% CI 0.720.99) when WHtR was approximately 0.47 (Supplemental Table 2 and Supplemental Figure 2).

### 3.4. Baseline BMI and WC with Respect to the Risk of Diabetes

The combined effects of baseline BMI and WC on the risk of diabetes are shown in Table 3. Compared to participants with normal weight and nonabdominal obesity, participants with normal weight and abdominal obesity (HR: 1.32; 95% CI:1.12, 1.56), participants with overweight and abdominal obesity (HR: 1.76; 95% CI: 1.56, 1.98) or nonabdominal obesity (HR: 1.32; 95% CI: 1.19, 1.47), and participants with general obesity and abdominal obesity (HR: 2.26; 95% CI: 1.98, 2.58) or nonabdominal obesity (HR: 1.52; 95% CI: 1.23, 1.88) were at a significantly increased risk for diabetes. However, participants who had a BMI < 18.5 kg/m^2^ were not significantly associated with a risk for diabetes, regardless of their abdominal obesity status at baseline.

### 3.5. Results of Subgroup Analyses and Sensitivity Analysis

**Figure 2:**
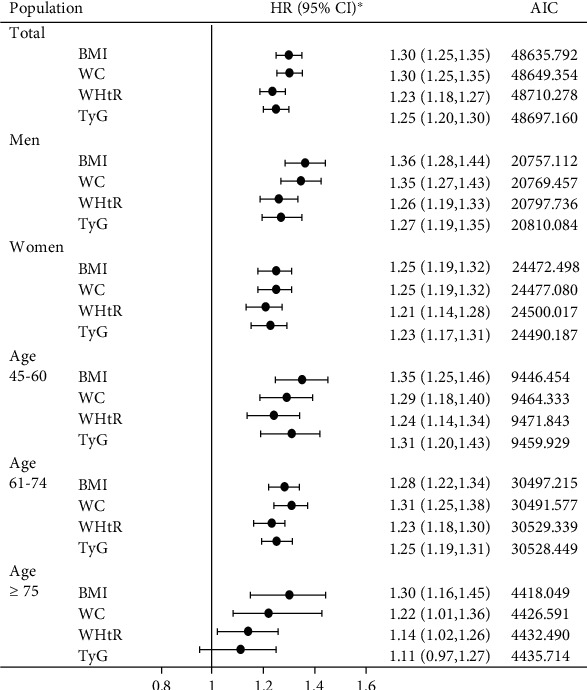
Association of T2DM and four indicators by sex and/or age groups. Asterisk adjusted for age, gender, marital status, smoking, alcohol consumption, physical activity, RHR, SBP, DBP, and TC levels. SBP: systolic blood pressure; DBP: diastolic blood pressure; RHR: resting heart rate; TC: total cholesterol; BMI: body mass index; WC: waist circumference.

To compare the strengths of the association of BMI, WC, WHtR, or TyG with diabetes, we calculated the HRs (95% CIs) of diabetes for 1 standard deviation (SD) increase in each of the four indicators, and the global goodness-of-fit of the models was assessed using AIC (Figure 2). Among the total population, BMI and WC were more significantly associated with diabetes risk than WHtR or TyG, with the multivariable-adjusted HRs (95% CIs) of diabetes being 1.30 (1.25–1.35) for BMI, 1.30 (1.25–1.35) for WC, 1.25 (1.20–1.30) for TyG, and 1.23 (1.18–1.27) for WHtR. In the subgroup analyses, similar trends were also observed in men, women, and those 61 years of age and older, and corresponding AIC values were lower for BMI and WC than for WHtR and TyG. Furthermore, men had a higher risk of developing diabetes than women, and HR estimates of diabetes risk were lower in participants aged ≥75 years than in participants aged <75 years. In the sensitivity analysis (Supplemental Table 1), the results were robust after excluding participants with IFG at baseline.

## 4. Discussion

In this retrospective analysis of a population-based sample of middle-aged and elderly adults, we found that BMI and WC were more strongly associated with diabetes than WHtR or TyG after controlling for a variety of potential confounders. In the subgroup analysis, the risk associated with diabetes appeared to decrease with age in all four measures, but BMI remained more strongly associated with diabetes risk among participants 75 years of age and older. BMI and WC were more strongly associated with diabetes in men than in women. In addition, combined analysis of BMI and WC greatly enhanced the strength of the association with diabetes risk. Compared to normal levels of BMI and WC, participants with high BMI and WC have the highest risk of developing diabetes.

Our study confirms previous findings that both general and abdominal obesity are strongly and independently correlated with the development of diabetes [3, 9, 18–22], and BMI and WC have stronger correlation with diabetes. Data from the British Regional Heart Study and the British Women's Heart and Health Study (3,519 men and 3,404 women) reported that BMI and WC were strongly and significantly associated with risk of T2DM, and yielded similar prediction in older men during 7 years of follow-up ^21^. Likewise, the health professionals follow-up study of 27,270 men aged 40–75 years indicated that BMI and WC showed similar associations related to the risk of T2DM [22]. Furthermore, Vazquez et al. conducted a meta-analysis of 32 studies and observed that the relative risks for diabetes were equivalent for standardized differences in BMI and WC [23]. In fact, there are also some inconsistent reports concerning which obesity indicators are more associated with diabetes and better predictors of the disease. Hou et al. demonstrated that WC and WHtR were more strongly associated with diabetes than BMI among 42,918 Chinese adults aged 20–88 years [24]. Data from health examinations of employees of the Kailuan Company City reported that WHtR and, to some degree WC, are the best predictors of T2DM in Tangshan subjects aged 18–85 years old during 2 years of follow-up [25]. The reason for these discrepancies may be that most of the studies included were conducted in populations with a wide range of ages. Taken together, these results suggest that anthropometric indicators associated with obesity are strongly associated with diabetes, particularly BMI and WC. In addition, we also found that it may be a better suggestion in reducing the risks of diabetes of middle-aged and elderly adults that being as lean as possible within the normal range of WC and WHtR in men and the appropriate cutoff points among women 72 cm for WC and 0.47 for WHtR. There were inconsistent with the results of Khader et al. in Jordanian with an average age of 43.8 [5], which showed that the optimal cut off points for the prevention of diabetes were 92 cm for WC and 0.60 for WHtR in women and 100 cm for WC and 0.57 for WHtR in men. Differences in age and ethnicity might explain the discrepancy in cut off values. Our study found that women were at higher risk of developing diabetes than men. Previous studies have found that body composition changes with age, including increased fat mass, decreased muscle mass, redistribution of fat tissue, and shrinkage of height [26]. In addition, women lost more height and stored more subcutaneous fat than men [27], which greatly increases the risk of diabetes. This may help explain why the recommended cutoffs were found only in women to some extent. In daily life, these simple and easily measured indicators can help middle-aged and elderly individuals implement early intervention measures, such as lifestyle modifications (balanced diet and appropriate physical exercise), to prevent the occurrence of diabetes.

A large number of studies have reported that TyG is predictive of the risk of incident diabetes [8, 11, 28], but it is still unclear whether the strength of the association between TyG and diabetes is better than easily measurable anthropometric markers, such as BMI, WC, and WHtR. Zhang et al. reported that among 5706 individuals (age ≥ 18 years), TyG, WC, and WHtR had no significant difference in predicting diabetes in men, while TyG had the best predictive effect in women [11]. However, BMI and WC were better predictors of diabetes risk in our study, while TyG and WHtR were less effective. A possible explanation of this phenomenon is that the TyG index is a biomarker associated with IR, but *β*-cell defects in insulin secretion may play a more important role in the pathogenesis of T2DM, and IR was not one of the most important factors in the elderly [29].

In agreement with our own results, previous studies identified gender differences in the association between obesity-related measures and T2DM [9, 19]. This gender difference may be caused by regional and whole-body muscle mass differences between men and women [30]. Furthermore, age may affect the risk of diabetes associated with the four measures examined in this study. This study found that HR estimates of diabetes risk were lower in individuals 75 years of age and older than in individuals under the age of 75, which was consistent with previous studies [26]. There are a number of potential explanations for this phenomenon. First, common anthropometric methods may not be good at quantifying body fat in older adults due to age-related changes in body composition, including redistribution of adipose tissue, loss of skeletal muscle mass, and height shrinkage [31]. Second, previous studies have found that in the etiology of diabetes, regional fat distribution such as visceral fat and intermuscular thigh fat is more important than the absolute amount of body fat [29, 32]. In addition, the correlation intensity between WC and visceral fat is inconsistent across different studies [33, 34], which may be due to differences in age, gender, race, overall degree of obesity, and cardiopulmonary health [35, 36]. In addition, the pathophysiological processes of diabetes in older adults may differ from those in young and middle-aged adults, and the effects of age observed may derive from selective survival in elderly individuals [26, 37].

We also tested the joint effects of abdominal adiposity (measured by WC) and overall body adiposity (measured by BMI). Similar to previous prospective population-based cohort studies [3, 18], we found that in joint analyses, the highest risk was observed in participants with high BMI combined with high WC. Moreover, overweight subjects with high WC had a significantly increased risk of diabetes, whereas obese subjects with low WC had no significantly increased risk of diabetes. The reason for this may be that, on the one hand, participants with a high BMI and high WC may have low muscle mass and thus a higher risk of developing diabetes, considering muscle tissue sensitivity to insulin [38]. On the other hand, increased WC indicates the accumulation of abdominal fat and further affects insulin metabolism by releasing free fatty acids, which may lead to insulin resistance in muscle and liver and impair *β*-cell function [38, 39]. Therefore, the simultaneous assessment of abdominal obesity and overall obesity improves risk prediction for diabetes.

The strengths of this study include a large sample size, long follow-up time, standardized measurements, and the use of an annual health examination dataset to some extent to avoid recall bias. Second, we compared the association between anthropometric and laboratory indicators and diabetes risk by gender and age, and further analyzed the joint effects of BMI and WC. Finally, we also conducted a sensitivity analysis to assess the robustness of the association between the four indicators and incident diabetes risk. However, this study has several limitations. First, the diagnosis of diabetes in this study was based on self-reporting, the use of antidiabetic medications, and fasting glucose measurements without 2-hour OGTT or glycated hemoglobin (HbA1c) levels, which may underestimate the incidence of diabetes. Second, the sample of this study is from the annual health examination dataset, which is primarily concentrated among middle-aged and elderly people and limits the universality of this study. Thirdly, even after adjusting for the major confounders in the analysis, there were still some remaining confounders that were not adjusted for, including unmeasured factors such as education and diet. Finally, because our study sample limits the generality of our results, the dose-response association should be considered with caution. In the future work, more studies may be needed to analyze the dose-response relationship between these indicators and diabetes by using restricted cubic splines, so as to verify this result and improve the accuracy of our study.

## 5. Conclusions

BMI and WC were more strongly associated with diabetes than either WHtR or TyG among a middle-aged and elderly population, suggesting that simple obesity-related indicators in daily life are simpler and more useful than laboratory indicators in predicting diabetes. We also found that an appropriate level may be chosen to minimize the risk of developing diabetes, with an approximate optimum of 72 cm for WC and 0.46 for WHtR in women. In addition, the use of BMI combined with WC significantly increased the degree of association with diabetes risk. This suggests that waist circumference should be measured in addition to BMI when assessing the risk for diabetes. Therefore, avoiding overweight and obesity at the same time as central obesity has important public health implications for the prevention of diabetes.

## Figures and Tables

**Table 1:** Baseline characteristics of the study population with and without diabetes.

Characteristics	Total (*n* = 41,242)	Nondiabetes (*n* = 38,770)	Diabetes (*n* = 2,472)	*P* value
Age (years)	63 (61–68)	63 (61–68)	63 (61–68)	0.42
Gender (%)				0.21
Men	19,552 (47.41)	18,410 (47.49)	1,142 (46.2)	
Women	21,690 (52.59)	20,360 (52.51)	1,330 (53.80)	
Marital status (%)				0.04
Living with partner	6,775 (16.43)	6,406 (16.52)	369 (14.93)	
Living without partner	34,467 (83.57)	32,364 (83.48)	2,103 (85.07)	
Smoking (%)				0.42
Never	34,446 (83.52)	32,367 (83.48)	2,079 (84.1)	
Current or previous	6,796 (16.48)	6,403 (16.52)	393 (15.90)	
Drinking (%)				0.36
Never	38,059 (92.28)	35,783 (92.30)	2,276 (92.07)	
Occasionally	2,386 (5.79)	2,247 (5.80)	139 (5.62)	
Daily	797 (1.93)	740 (1.91)	57 (2.31)	
Physicial activity (%)				0.46
Never	29,733 (72.09)	27,978 (72.16)	1,755 (71.00)	
Occasionally	2,260 (5.48)	2,109 (5.44)	151 (6.11)	
More than once a week	1,766 (4.28)	1,657 (4.27)	109 (4.41)	
Daily	7,483 (18.14)	7,026 (18.12)	457 (18.49)	
SBP (mmHg)	130.00 (120.00–140.00)	130.00 (120.00–140.00)	130.00 (120.00–142.00)	0.001
DBP (mmHg)	80.00 (71.00–85.00)	80.00 (71.00–85.00)	80.00 (73.00–85.00)	0.001
RHR	73.00 (68.00–78.00)	73.00 (68.00–78.00)	74.00 (69.00–79.00)	<0.001
FPG (mmol/L)	5.12 (4.66–5.60)	5.11 (4.65–5.59)	5.36 (4.80–5.90)	<0.001
TC (mmol/L)	4.69 (4.13–5.30)	4.69 (4.13–5.30)	4.69 (4.16–5.33)	0.55
TG (mmol/L)	1.21 (0.89–1.60)	1.21 (0.89–1.59)	1.30 (0.95–1.70)	<0.001
BMI (kg/m2)	23.94 (22.21–26.23)	23.88 (22.15–26.17)	24.77 (22.94–27.14)	<0.001

WC (cm) 83.00 (77.00–89.00) 83.00 (77.00–89.00) 84.00 (78.00–90.00) <0.001. WHtR 0.52 (0.48–0.56) 0.52 (0.48–0.56) 0.52 (0.48–0.56) <0.001. TyG index 8.50 (8.16–8.80) 8.50 (8.16–8.79) 8.61 (8.27–8.93) <0.001.

**Table 2:** Risk of incident diabetes by baseline BMI, WC, WHtR, and TyG.

		No. of cases	No. of person-years	Incidence rate^†^	HRs (95% CIs)		
					Model 1^‡^	Model 2^§^	Model 3^¶^
BMI, kg/m2	<18.5	23	3,827	6.01	0.64 (0.42,0.97)	0.68 (0.45,1.03)	0.69 (0.46,1.04)
	18.5–23.9	975	103,514	9.42	1.00 (ref)	1.00 (ref)	1.00 (ref)
	24–27.9	1,032	74,683	13.82	1.49 (1.37,1.63)	1.43 (1.31,1.56)	1.41 (1.29,1.54)
	≥28.0	442	23,746	18.61	2.09 (1.87,2.34)	1.98 (1.77,2.22)	1.92 (1.71,2.16)
*P* trend					<0.001	<0.001	<0.001
WC, cm	<77.00	549	58,488	9.39	1.00 (ref)	1.00 (ref)	1.00 (ref)
	77.00–81.99	566	51,298	11.03	1.22 (1.09,1.38)	1.20 (1.07,1.35)	1.19 (1.06,1.34)
	82.00–88.99	607	50,998	11.90	1.40 (1.25,1.57)	1.35 (1.20,1.52)	1.34 (1.19,1.51)
	≥89.00	750	44,986	16.67	2.15 (1.92,2.40)	2.04 (1.82,2.29)	1.99 (1.78,2.23)
*P* trend					<0.001	<0.001	<0.001
WC, cm	<85 (women)/90 (men)	1,499	142,907	10.49	1.00 (ref)	1.00 (ref)	1.00 (ref)
	≥85 (women)/90 (men)	973	62,863	15.48	1.67 (1.54,1.81)	1.61 (1.48,1.75)	1.57 (1.45,1.71)
WHtR	<0.47	583	57,475	10.14	1.00 (ref)	1.00 (ref)	1.00 (ref)
	0.47–0.51	583	53,243	10.95	1.13 (1.01,1.27)	1.11 (0.99,1.24)	1.10 (0.98,1.24)
	0.52–0.55	632	49,585	12.75	1.37 (1.23,1.54)	1.34 (1.20,1.50)	1.33 (1.18,1.49)
	≥0.56	674	45,467	14.82	1.73 (1.55,1.93)	1.70 (1.52,1.91)	1.65 (1.47,1.86)
*P* trend					<0.001	<0.001	<0.001
WHtR	<0.5	871	85,788	10.15	1.00 (ref)	1.00 (ref)	1.00 (ref)
	≥0.5	1,601	119,982	13.34	1.45 (1.33,1.57)	1.42 (1.30,1.54)	1.39 (1.28,1.51)
TyG	<8.16	465	49,261	9.44	1.00 (ref)	1.00 (ref)	1.00 (ref)
	8.16–8.50	545	51,456	10.59	1.08 (0.95,1.22)	1.09 (0.96,1.23)	1.10 (0.97,1.25)
	8.51–8.80	645	54,040	11.94	1.20 (1.06,1.35)	1.21 (1.07,1.36)	1.23 (1.09,1.39)
	≥8.81	817	51,013	16.02	1.61 (1.44,1.81)	1.62 (1.44,1.82)	1.66 (1.47,1.87)
*P* trend					<0.001	<0.001	<0.001

^†^ Per 1,000 person-years. ^‡^ Unadjusted. ^§^ Adjusted for variables in b as well as age, gender, marital status, smoking, alcohol consumption and physical activity. ¶ Adjusted for variables in c as well as RHR, SBP, DBP, and TC levels. SBP: systolic blood pressure; DBP: diastolic blood pressure; RHR: resting heart rate; TC: total cholesterol; BMI: body mass index; WC: waist circumference; WHtR: waist-to-height ratio; TyG: triglyceride glucose index.

**Table 3:** HRs of diabetes based on different levels of baseline BMI and WC in middle-aged and elderly Chinese people.

	Baseline WC, cm	
	*<*85 (women)/90 (men)	≥85 (women)/90 (men)
BMI < 18.5 kg/m2		
No. of cases	22	1
No. of person-years	3,617	210
Incidence rate^†^	6.08	4.76
HRs (95% CIs) ^‡^	0.73 (0.48,1.12)	0.57 (0.08,4.04)
BMI ≥ 18.5 to <24 kg/m2		
No. of cases	803	172
No. of person-years	88,710	14,804
Incidence rate†	9.05	11.62
HRs (95% CIs) ^‡^	1.00 (ref)	1.32 (1.12,1.56)
BMI ≥ 24 to <28 kg/m2		
No. of cases	576	456
No. of person-years	44,603	30,080
Incidence rate^†^	12.91	15.16
HRs (95% CIs) ^‡^	1.32 (1.19,1.47)	1.76 (1.56,1.98)
BMI ≥ 28 kg/m2		
No. of cases	98	344
No. of person-years	5,977	17,769
Incidence rate^†^	16.40	19.36
HRs (95% CIs) ^‡^	1.52 (1.23,1.88)	2.26 (1.98,2.58)

^†^ Per 1,000 person-years. ^‡^ Adjusted for age, gender, marital status, smoking, alcohol consumption, physical activity, RHR, SBP, DBP, and TC levels. SBP: systolic blood pressure; DBP: diastolic blood pressure; RHR: resting heart rate; TC: total cholesterol; BMI: body mass index; WC: waist circumference.

## Data Availability

The data used to support the findings of this study cannot be disclosed due to confidentiality agreement.

## Supplementary Materials

Supplementary Materials Figure S1: flow diagram of participant selection. Table S1: risk of incident diabetes for participants without impaired FPG level at baseline. Table S2: Nonlinearity tests of obesity indicators for outcomes. Figure S2: dose-response relationship between three indicators (waist circumference, waist to height ratio, and triglyceride glucose index) and T2DM risk in women (A, C, and E) and men (B, D, and F).
