# Supplementary material for: Stronger Associations of Body Mass Index and Waist Circumference with Diabetes than Waist-Height Ratio and Triglyceride Glucose Index in the Middle-Aged and Elderly Population: A Retrospective Cohort Study
Source: J Diabetes Res. 2022 Feb 26;2022:9982390. doi: 10.1155/2022/9982390 (PMC8898128; doi:10.1155/2022/9982390)
Supplement: Supplementary Materials — Figure S1: flow diagram of participant selection. Table S1: risk of incident diabetes for participants without impaired FPG level at baseline. Table S2: Nonlinearity tests of obesity indicators for outcomes. Figure S2: dose-response relationship between three indicators (waist circumference, waist to height ratio, and triglyceride glucose index) and T2DM risk in women (A, C, and E) and men (B, D, and F). [file 9982390.f1.docx]

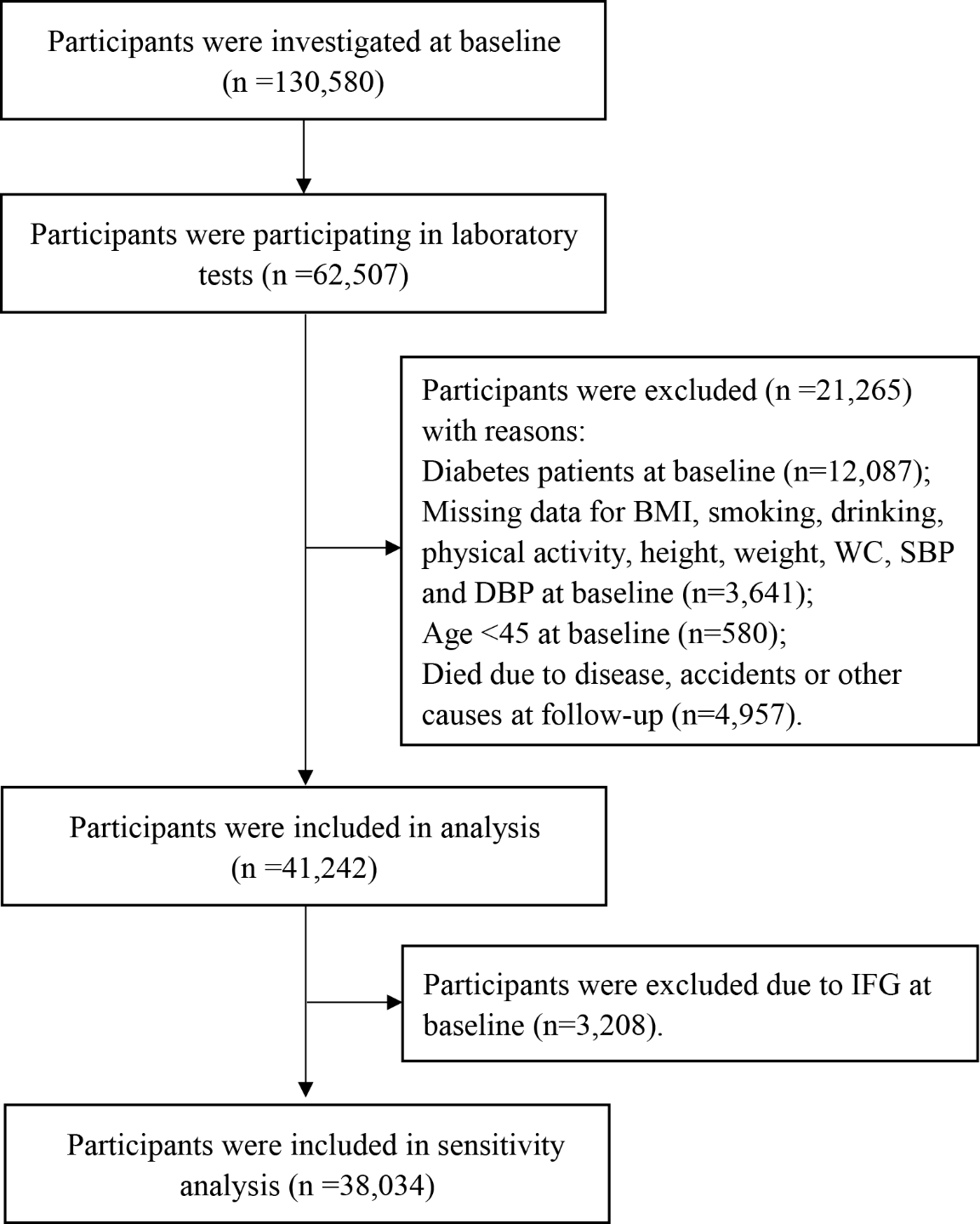


**Supplemental Figure 1 Flow diagram of participant selection**

**Supplemental Table 1** Risk of incident diabetes for participants without impaired FPG level at baseline

|  |  | No. of cases | No. of person-years | Incidence rate^†^ | HRs (95% CIs) | | |
| --- | --- | --- | --- | --- | --- | --- | --- |
|  |  |  |  |  | Model 1^‡^ | Model 2^§^ | Model 3^¶^ |
| BMI | <18.5 | 21 | 3,610 | 5.82 | 0.68 (0.44,1.05) | 0.72 (0.47,1.11) | 0.72 (0.47,1.12) |
|  | 18.5-23.9 | 827 | 96,767 | 8.55 | 1.00 (ref) | 1.00 (ref) | 1.00 (ref) |
|  | 24-27.9 | 846 | 68,434 | 12.36 | 1.48 (1.34,1.62) | 1.42 (1.29,1.56) | 1.41 (1.28,1.55) |
|  | ≥28.0 | 339 | 21,282 | 15.93 | 1.98 (1.74,2.24) | 1.88 (1.65,2.13) | 1.85 (1.63,2.11) |
| *P* _trend_ | |  |  |  | <0.001 | <0.001 | <0.001 |
| WC | <76.00 | 478 | 54,602 | 8.75 | 1.00 (ref) | 1.00 (ref) | 1.00 (ref) |
|  | 76.00-81.99 | 485 | 47,713 | 10.16 | 1.21 (1.07,1.38) | 1.20 (1.05,1.36) | 1.19 (1.05,1.36) |
|  | 82.00-88.99 | 495 | 46,975 | 10.54 | 1.34 (1.18,1.52) | 1.30 (1.15,1.48) | 1.30 (1.14,1.48) |
|  | ≥89.00 | 575 | 40803 | 14.09 | 1.97 (1.75,2.23) | 1.90 (1.67,2.15) | 1.88 (1.66,2.13) |
| *P* _trend_ | |  |  |  | <0.001 | <0.001 | <0.001 |
| WHtR | <0.47 | 509 | 53,638 | 9.49 | 1.00 (ref) | 1.00 (ref) | 1.00 (ref) |
|  | 0.47-0.51 | 486 | 49,335 | 9.85 | 1.09 (0.97,1.24) | 1.07 (0.95,1.21) | 1.07 (0.95,1.21) |
|  | 0.52-0.55 | 512 | 44,974 | 11.38 | 1.32 (1.17,1.50) | 1.30 (1.15,1.47) | 1.30 (1.15,1.47) |
|  | ≥0.56 | 526 | 42,146 | 12.48 | 1.57 (1.39,1.78) | 1.56 (1.37,1.76) | 1.54 (1.36,1.75) |
| *P* _trend_ | |  |  |  | <0.001 | <0.001 | <0.001 |
| TyG | <8.14 | 410 | 45,407 | 9.03 | 1.00 (ref) | 1.00 (ref) | 1.00 (ref) |
|  | 8.14-8.47 | 470 | 47,399 | 9.92 | 1.05(0.92,1.20) | 1.06(0.93,1.21) | 1.07(0.93,1.22) |
|  | 8.48-8.76 | 534 | 49,759 | 10.73 | 1.12(0.98,1.27) | 1.12(0.99,1.28) | 1.15(1.00,1.31) |
|  | ≥8.77 | 619 | 47,528 | 13.02 | 1.35(1.19,1.53) | 1.35(1.19,1.54) | 1.39(1.22,1.58) |
| *P* _trend_ | |  |  |  | <0.001 | <0.001 | <0.001 |

^†^ Per 1,000 person-years

^‡^ Unadjusted

^§^ Adjusted for variables in b as well as age, gender, marital status, smoking, alcohol consumption and physical activity

^¶^ Adjusted for variables in c as well as RHR, SBP, DBP and TC levels

SBP, systolic blood pressure; DBP, diastolic blood pressure; RHR, resting heart rate; TC, total cholesterol; BMI, body mass index; WC, waist circumference.

**Supplemental Table 2 Non-linearity tests of obesity indicators for outcomes.**

|  |  | reference | *P* for overall association | *P* for nonlinearity |
| --- | --- | --- | --- | --- |
| total | BMI | 23.94 | <0.001 | 0.408 |
|  | WC | 83.00 | <0.001 | 0.001 |
|  | WHtR | 0.52 | <0.001 | 0.002 |
|  | TyG | 8.52 | <0.001 | 0.001 |
| men | WC | 84.00 | <0.001 | 0.376 |
|  | WHtR | 0.51 | <0.001 | 0.416 |
|  | TyG | 8.43 | <0.001 | 0.296 |
| women | WC | 81.00 | <0.001 | 0.003 |
|  | WHtR | 0.53 | <0.001 | 0.005 |
|  | TyG | 8.56 | <0.001 | 0.001 |

BMI, body mass index; WC, waist circumference; WHtR, waist-to-height ratio; TyG, triglyceride glucose index.


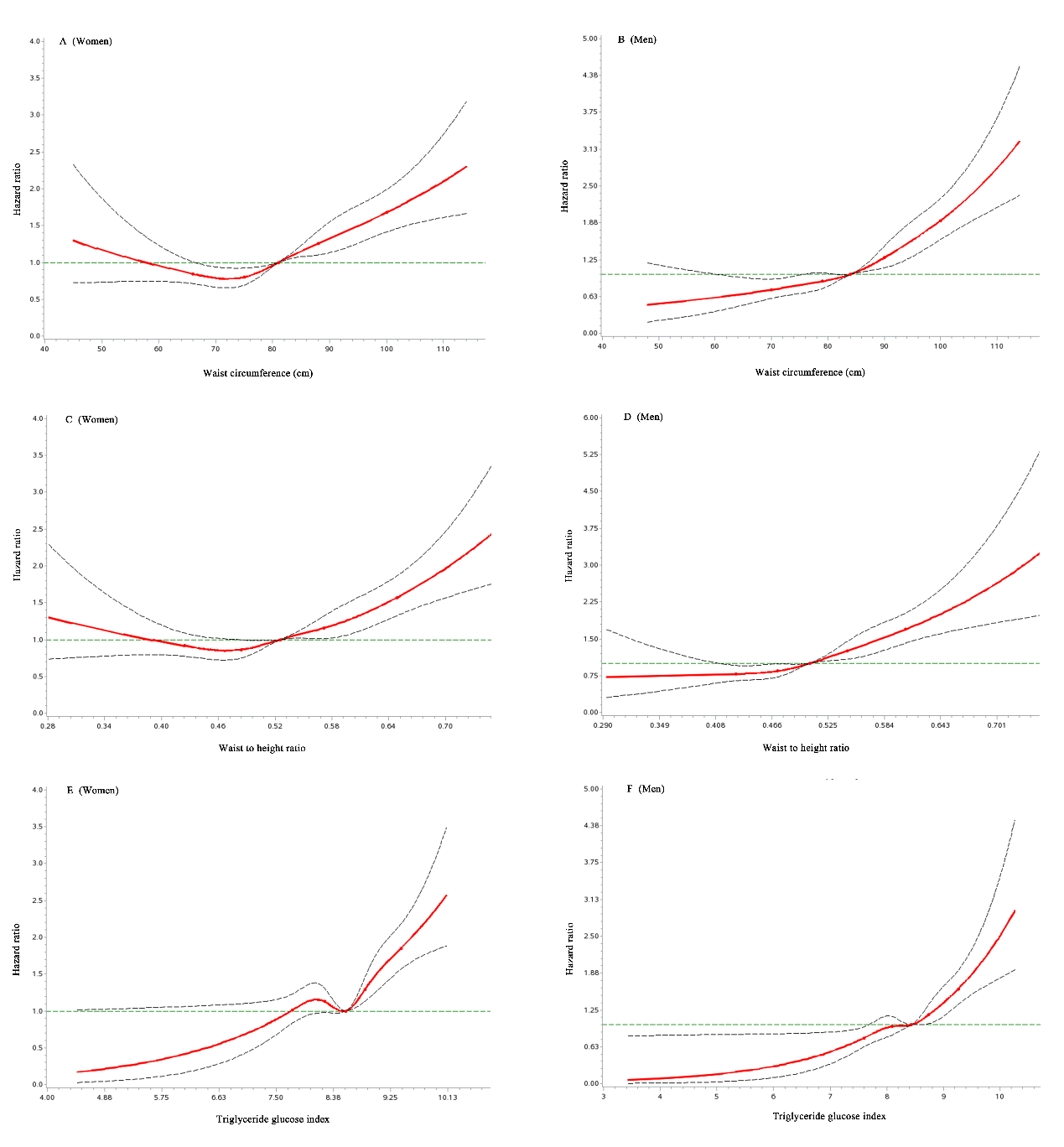


**Supplemental Figure 2** Dose–response relationship between three indicators (waist circumference, waist to height ratio and triglyceride glucose index) and T2DM risk in women (A, C and E) and men (B, D, and F). The circles represent the points (5, 25, 50, 75, 95 percentiles) where the nodes were placed. The region between the two dotted lines represents the 95% confidence intervals. The model was adjusted for age, gender, marital status, smoking, drinking, physical activity, RHR, RHR, SBP, DBP and TC levels. Values were trimmed at less than 1st percentile and greater than 99th percentile of each indicator. SBP, systolic blood pressure; DBP, diastolic blood pressure; RHR, resting heart rate; TC, total cholesterol; WC, waist circumference.
